# Commentary: Immunogenomic characteristics and prognostic implications of terminally exhausted CD8+ T cells in colorectal cancers

**DOI:** 10.3389/fimmu.2025.1654000

**Published:** 2025-08-13

**Authors:** Wenjiang Wu, Yingjie Liu, Peishan Yu, Linchong Yu

**Affiliations:** Shenzhen Hospital (Futian) of Guangzhou University of Chinese Medicine, Shenzhen, Guangdong, China

**Keywords:** CRC (colorectal cancer), CD8+T cell exhaustion, spatial biology, sample bias, ICD (immunogenic cell death)

## 1 Introduction

The study by Lee et al. addresses a timely question: how T-cell exhaustion heterogeneity influences CRC outcomes, a heterogeneity now recognized to involve distinct functional subsets defined by co-inhibitory receptor combinations (1).”. Ttex (TCF1-PD1+CD8+) and progenitor exhausted T cells (Tpex) were quantified via mIF in tissue microarrays (TMAs), integrated with genomic data (MSI/TMB; 40-gene panel). The discovery that Ttex enrichment predicts survival in oxaliplatin-treated patients is compelling, yet three key issues merit deeper scrutiny: sample representativeness, technical validation gaps, and mechanistic ambiguity.

## 2 Methodological and translational challenges

### 2.1 ​Spatial biology and sample bias​

The TMA design—two 1-mm cores each from tumor center (TC) and invasive margin (IM)—risks underrepresenting spatial heterogeneity. Tertiary lymphoid structures (TLS), critical niches for Tpex, were excluded, potentially skewing exhaustion subset distributions. This approach directly contradicts spatial analyses demonstrating TLS as critical reservoirs for Tpex (2, 3).”While IM CD8+ density showed weak correlation between TMA and whole-slide imaging (WSI; ρ=0.34), TC correlations were stronger (ρ=0.60). This inconsistency questions TMA’s reliability for spatial immune mapping. Future studies should prioritize WSI-based mIF to capture TLS dynamics.

### 2.2 ​Phenotypic oversimplification​

Ttex was defined solely as TCF1-PD1+CD8+, overlooking co-inhibitory receptors (e.g., TIM-3, LAG-3) that define terminal exhaustion. Recent single-cell studies (1, 4) show Ttex can retain cytotoxic function if specific receptor combinations are present. Recent single-cell evidence confirms that Ttex with specific receptor profiles (e.g., PD-1+TCF1-TIM-3-) retain cytotoxic function (1), while those co-expressing ≥3 inhibitors exhibit true terminal exhaustion. Incorporating ≥3 exhaustion markers would clarify whether “prognostic” Ttex subsets exhibit functional resilience. Specifically, Lee et al.’s own data (Lee et al.’s Figure 4) shows worse survival in ‘Teff-high’ groups, implying that cytotoxic potential alone cannot explain prognosis—reinforcing the need for multi-receptor phenotyping.

### 2.3 ​Mechanistic disconnect​

The link between Ttex and oxaliplatin response remains correlative. Oxaliplatin induces immunogenic cell death (ICD), potentially activating Ttex. However, *no functional assays* (e.g., IFN-γ secretion or tumor-killing capacity) support this. Oxaliplatin-induced immunogenic cell death (ICD) is known to activate T-cell responses via HMGB1 release (5), yet Ttex functionality under ICD remains unproven. Validating Ttex functionality in ICD models (e.g., CRC organoids co-cultured with Ttex) is essential. Three mechanistic gaps persist: (i) No correlation between Ttex density and ICD biomarkers (e.g., HMGB1/ATP). (ii) Absence of Ttex functional data post-oxaliplatin exposure (6). (iii) Paradoxical survival benefit in CD8-low/Ttex-high tumors despite presumed dysfunction.

## 3 Discussion

Lee et al. provide foundational evidence that Ttex may refine CRC prognostication, but translating this into clinical utility requires three synergistic advances: spatially resolved whole-slide mIF must replace TMAs to incorporate tertiary lymphoid structures and ensure reliable invasive margin analysis, multi-parametric exhaustion signatures integrating ≥3 markers (e.g., PD-1+TCF1−TIM-3+) should be deployed via hyperplexed platforms like CODEX to distinguish functional subsets, and Ttex responsiveness must be functionally interrogated in immunogenic cell death models with/without anti-PD-1 to identify therapeutic synergies—particularly as the prognostic value of Ttex in CD8-low tumors holds revolutionary potential for adjuvant stratification, making validation in biomarker-driven trials such as FOxTROT with serial pre/post-chemotherapy mapping a critical next step.
